# Validity of the Manchester Triage System in emergency care: A prospective observational study

**DOI:** 10.1371/journal.pone.0170811

**Published:** 2017-02-02

**Authors:** Joany M. Zachariasse, Nienke Seiger, Pleunie P. M. Rood, Claudio F. Alves, Paulo Freitas, Frank J. Smit, Gert R. Roukema, Henriëtte A. Moll

**Affiliations:** 1 Department of General Paediatrics, Erasmus MC- Sophia Children’s Hospital, Rotterdam, The Netherlands; 2 Department of Emergency Medicine, Erasmus MC, Rotterdam, The Netherlands; 3 Department of Paediatrics, Emergency Unit, Hospital Professor Doutor Fernando da Fonseca, Amadora, Portugal; 4 Intensive Care Unit, Hospital Professor Doutor Fernando da Fonseca, Amadora, Lisbon, Portugal; 5 Department of Paediatrics, Maasstad Hospital, Rotterdam, The Netherlands; 6 Department of Surgery, Maasstad Hospital, Rotterdam, The Netherlands; Yokohama City University, JAPAN

## Abstract

**Objectives:**

To determine the validity of the Manchester Triage System (MTS) in emergency care for the general population of patients attending the emergency department, for children and elderly, and for commonly used MTS flowcharts and discriminators across three different emergency care settings.

**Methods:**

This was a prospective observational study in three European emergency departments. All consecutive patients attending the emergency department during a 1-year study period (2010–2012) were included. Validity of the MTS was assessed by comparing MTS urgency as determined by triage nurses with patient urgency according to a predefined 3-category reference standard as proxy for true patient urgency.

**Results:**

288,663 patients were included in the analysis. Sensitivity of the MTS in the three hospitals ranged from 0.47 (95%CI 0.44–0.49) to 0.87 (95%CI 0.85–0.90), and specificity from 0.84 (95%CI 0.84–0.84) to 0.94 (95%CI 0.94–0.94) for the triage of adult patients. In children, sensitivity ranged from 0.65 (95%CI 0.61–0.70) to 0.83 (95%CI 0.79–0.87), and specificity from 0.83 (95%CI 0.82–0.83) to 0.89 (95%CI 0.88–0.90). The diagnostic odds ratio ranged from 13.5 (95%CI 12.1–15.0) to 35.3 (95%CI 28.4–43.9) in adults and from 9.8 (95%CI 6.7–14.5) to 23.8 (95%CI 17.7–32.0) in children, and was lowest in the youngest patients in 2 out of 3 settings and in the oldest patients in all settings. Performance varied considerably between the different emergency departments.

**Conclusions:**

Validity of the MTS in emergency care is moderate to good, with lowest performance in the young and elderly patients. Future studies on the validity of triage systems should be restricted to large, multicenter studies to define modifications and improve generalizability of the findings.

## Introduction

Triage at the emergency department (ED) aims to prioritize patients when clinical demand exceeds capacity.[1] As the burden on emergency departments worldwide is steadily increasing, triage remains a fundamental intervention to manage patient flow safely and to ensure that patients who need immediate medical attention are timely treated, particularly in case of overcrowding.[2–5]

The Manchester Triage System (MTS) is one of the most commonly used triage systems in Europe.[6] It enables nurses to assign a clinical priority to patients, based on presenting signs and symptoms, without making any assumption about the underlying diagnosis. The MTS allocates patients to one out of five urgency categories, which determine the maximum time to first contact with a physician. Despite its widespread implementation, validity of the MTS remains uncertain. Previous research consists of single center studies,[7–10] studies restricted to certain age groups or specific medical conditions,[11–15] and studies analyzing validity by trends in resource use or hospitalisation.[7, 8, 10, 16] To date, no study has evaluated performance of the MTS in a large, heterogeneous cohort of patients, at different emergency departments, and with a reference standard that is independent of triage, correlated to severity of illness, and applicable to patients with a wide range of presenting problems.

The aim of this study is to determine the performance of the Manchester Triage System for the general population of patients attending the emergency department and specifically for children and elderly, the most vulnerable groups of patients. Moreover, we aim to evaluate the performance of the most commonly used MTS flowcharts and discriminators. Knowledge about the validity of MTS can provide insight in its performance, it enables the comparison with other triage systems and it can support targeted modifications for improvement.

## Methods

### Study design

The study is based on a multicenter prospective observational cohort of patients presenting to emergency departments in three different practice settings. Data collected during routine care was automatically extracted from electronic medical health records. The validity of the MTS was assessed by comparing MTS urgency as determined by triage nurses with patient urgency according to a predefined 3-category reference standard. Moreover, we assessed validity by the ability of the MTS high urgency categories to identify patients requiring Intensive Care Unit (ICU) admission or patients that died at the ED. We evaluated the performance of the MTS for different age groups and for the most commonly used flowcharts and discriminators. The study was approved by the institutional review boards of all participating institutions and the need for written informed consent from the participants was waived.

### Study population and setting

All consecutive patients attending the emergency departments of the Erasmus MC, Rotterdam, the Netherlands (July 2010 to July 2011); Maasstad Hospital, Rotterdam, the Netherlands (July 2011 to July 2012); and Hospital Professor Doutor Fernando da Fonseca (hereafter: Hospital Fernando Fonseca), Lisbon, Portugal (September 2011 to September 2012) were included in the study. Before the study period, all hospitals had two to five years of experience with the MTS.

Erasmus MC is an inner-city university hospital and tertiary care referral and trauma centre, with an ED receiving approximately 24,000 adults and 7,000 children a year. The ED delivers general emergency medicine, but as a tertiary care facility is specialized in complex care. Because the Netherlands has a strong system of primary care, and general practitioners act as gatekeepers, the proportion of low urgent patients is relatively small.

Maasstad Hospital is an inner-city teaching hospital with a mixed emergency department for adult and pediatric patients receiving approximately 38,000 patients a year. The ED delivers general emergency and trauma care. Similarly to the Erasmus, the proportion of low urgent patients is relatively small, because patients with minor complaints are usually seen by the GP or GP cooperative.

Hospital Fernando Fonseca is an inner-city community hospital with an annual census of approximately 190,000 adults and 60,000 children. The hospital delivers general emergency care and trauma care except neuro-surgery. Primary care is often not accessible for patients, and the ED is frequented by a large proportion of patients with minor complaints.

Therefore, settings with a different case-mix contributed to the study.

### Manchester Triage System

The MTS is a triage algorithm that consists of 52 flowcharts, covering patients’ chief signs and symptoms such as “Headache”, “Shortness of breath” and “Wounds”. Each flowchart in turn consists of additional signs and symptoms named discriminators, such as “Airway compromise”, “Severe pain” or “Persistent vomiting”, which are ranked by priority. General discriminators appear throughout the different charts while specific discriminators apply to small groups of presentations. Triage nurses select for each patient the most appropriate flowchart and consequently gather information on the discriminators from top to bottom. Selection of a discriminator allocates the patient to the related urgency category, ranging from “immediate” (0 minutes maximum waiting time) to “non-urgent” (240 minutes maximum waiting time). A discriminator will lead to the same urgency level, regardless of the flowchart used, increasing the ease of use and the interrater reliability.

In all three hospitals, trained nurses perform triage with a computerized triage application. Both Erasmus MC and Maasstad Hospital use the official Dutch translation of the second edition of the MTS.[17, 18] In the Erasmus MC, some specific modifications for children are implemented based on previous research.[19] The main difference includes a modification for children with fever. The Hospital Fernando Fonseca uses the official Portuguese translation of the second edition of the MTS which includes already some of the modifications implemented in the third edition of the MTS.[20] These differences consist of adaptations for children with fever, and the addition of a small number of extra discriminators. Details on the different versions of the MTS used in the study are provided in the supporting information (S1 Table).

### 3- category reference standard

Before the study started, a reference standard as proxy for patients’ true urgency was developed. We defined several requirements for our reference standard. It had to be a good proxy for patient urgency, independent of triage, be applied to individual patients with a wide range of problems, contain objective items that could be compared between settings, and identify at least 3 urgency levels to allow for evaluation of modifications.[21] First, we performed a literature review to identify currently used reference standards for triage. None of the reference standards fulfilled all our requirements.[22] Therefore, we composed an expert panel, consisting of a neurologist, a surgeon specialized in traumatology, an internist specialized in intensive care, a cardiologists, an emergency physician and a pediatrician. In an evaluation meeting, the panel discussed the individual reference standards and combined a selection of the most relevant items into a multilevel reference standard.

The final reference standard, as presented in Table 1, consisted of three urgency categories based on a combination of vital signs, treatment at the emergency department and patient disposition. Vital signs were measured at discretion of the nurse, and therefore not all patients had a complete set of vital signs recorded. If vital signs were not documented, they were assumed to be normal, which is in agreement with clinical experience. The low number of vital signs documented in the least urgent patients (e.g. heart rate was measured in 74% of patients in reference category 1 versus 36% in reference category 3) and the co-occurrence of missing vital signs in the same patients made it impossible to perform multiple imputation to handle the missing data. However, these findings also support our assumption that patients with missing vital signs in the absence of any other positive reference standard item are unlikely to be urgent.

**Table 1:** 3-category reference standard as proxy for true patient urgency.

Category	Corresponding MTS category	Maximum waiting time (minutes)	Items adults	Items children
R1	Immediate and Very urgent	0–10	Abnormal vital signs as defined by a modified early warning score ≥5 [23]	Abnormal vital signs according to a previously used reference standard,[11] based on the pediatric risk of mortality score (PRISM III) [24]
			Level of consciousness reacting to pain or unresponsive	Level of consciousness reacting to pain or unresponsive
			Mortality at the ED, ICU or high care admission*	Mortality at the ED or ICU admission
			Emergency surgery <4hours after arrival, including cardiac catheterization and endovascular aortic repair procedures *	
R2	Urgent	60	IV medication, fluids or nebulizers at the ED Hospitalization	IV medication, fluids or nebulizers at the ED Hospitalization
R3	Standard and Non-urgent	120–240	None of the above	None of the above

*Patients at hospital Fernando Fonseca do not have information on high care admission or emergency surgery available

### Data collection

Data on patient characteristics, triage, vital signs, resource utilization, admission to hospital, and follow-up are routinely documented in all hospitals and were automatically extracted from the electronic hospital information systems. Trained medical students entered data that was only available on paper emergency department forms in a separate database, blinded to MTS urgency, using SPSS Data entry version 4.0.

### Data analysis

First, validity of the MTS high urgency categories (“immediate” and “very urgent”) was assessed for the identification of patients requiring ICU admission, including the patients that died at the ED. We included ICU admission and death as a separate reference standard, because it has a strong correlation with patient urgency and is relatively independent of the clinical setting.

Second, for each individual patient, a reference standard category was determined, based on the 3-category reference standard. We assessed validity of the MTS by comparing the allocated MTS urgency category with the reference urgency category.

Validity was assessed by the proportion of correctly triaged, undertriaged and overtriaged patients and by the different diagnostic performance measures sensitivity, specificity, positive and negative likelihood ratio and diagnostic odds ratio. Undertriage was defined as the proportion of patients who were allocated to a lower MTS urgency category than the reference category and overtriage as the proportion of patients allocated to a higher MTS urgency category than the reference category. To calculate the diagnostic performance measures, we dichotomized the MTS and the reference standard into high (MTS category “immediate” and “very urgent”; reference category 1) and low urgency (MTS category “urgent”, “standard” and “non-urgent”; reference category 2 and 3). Sensitivity analyses were performed to assess the impact on MTS performance of the modifications for children with fever that were adopted in the Erasmus MC and Hospital Fernando Fonseca.[19,20] We did not assess the effect of other modifications because these were all together only applied in 1.9% of patients.

The MTS was validated for different subgroups based on age, and we determined five clinically relevant age groups: <1 year, 1 to 16 years, 16 to 65 years, 65 to 80 years and ≥80 years. Finally, we assessed validity of the most commonly used flowcharts and general discriminators in adult patients. Discriminators were grouped into the hemorrhage, consciousness and temperature discriminators.[6] We compared performance of these flowcharts and discriminators in adult patients with performance in the subgroup of patients aged 65 and older.

Analyses were performed using SPSS software (version 20.0). Diagnostic performance measures with 95% confidence intervals were calculated with the VassarStats website (http://statline.cbs.nl/statweb).

## Results

### Characteristics of study subjects

During the study period 306,090 patients attended the emergency department of one of the three hospitals. After the exclusion of patients with incomplete information on triage or reference standard items, 288,663 patients (94.3%) were available for analysis: 25,583 from the Erasmus MC, 32,532 from the Maasstad Hospital and 230,548 from Hospital Fernando Fonseca (S1 Fig). The Erasmus MC has a relatively high percentage of missing MTS urgency, which can be explained by the absence of triage nurses during night shifts at the start of the study. Since these missing values are expected to be at random, we used a complete case approach.

Hospital Fernando Fonseca has the largest caseload while the two Dutch hospitals have the most severe case-mix with a larger percentage of hospital and ICU admitted patients. Further characteristics of the study populations are presented in Table 2.

**Table 2:** Characteristics of the study population.

	Erasmus MC (n = 25,583)	Maasstad (n = 32,532)	Fernando Fonseca (n = 230,548)
**Age categories, n (%)**			
0–16 years	6185 (24.2)	7032 (21.6)	52,843 (22.9)
16–65 years	15,980 (62.5)	18,226 (56.0)	127,562 (55.3)
≥65 years	3418 (13.4)	7274 (22.4)	50,143 (21.7)
**Gender, n (%)**			
Male	14,611 (57.1)	16,600 (51.0)	99,406 (43.1)
Female	10,972 (42.9)	15,932 (49.0)	131,142 (56.9)
Presenting problem, n (%)			
Cardiac	1780 (7.0)	993 (3.1)	14,185 (6.2)
Dermatological	2960 (11.6)	3969 (12.2)	22,251 (9.7)
Ear, Nose and Throat	796 (3.1)	475 (1.5)	20,236 (8.8)
Gastrointestinal	3109 (12.2)	4681 (14.4)	29,101 (12.6)
Neurologic or psychiatric	2644 (10.3)	1769 (5.4)	16,217 (7.0)
Respiratory	1631 (6.4)	3079 (9.5)	21,955 (9.5)
Trauma or muscular	7536 (29.5)	11,689 (35.9)	53,711 (23.3)
General malaise	3304 (12.9)	3463 (10.6)	16,869 (7.3)
Uro- or gynaecological	752 (2.9)	620 (1.9)	18,422 (8.0)
Other or unknown	1071 (4.2)	1794 (5.5)	17,601 (7.6)
**MTS urgency, n (%)**			
Immediate	432 (1.7)	208 (0.6)	1365 (0.6)
Very urgent	2425 (9.5)	5075 (15.6)	37,502 (16.3)
Urgent	11,516 (45.0)	16,811 (51.7)	76,777 (33.3)
Standard	11,016 (43.1)	10,332 (31.8)	109,956 (47.7)
Non-urgent	194 (0.8)	106 (0.3)	4948 (2.1)
**Disposition, n (%)**			
Hospital admission	6914 (27.0)	9472 (29.1)	26,832 (11.6)
ICU admission	438 (1.7)	245 (0.8)	461 (0.2)
Mortality at the ED	43 (0.2)	32 (0.1)	74 (<0.1)

### Overall validity of the MTS

Sensitivity of the MTS to identify patients that died at the ED or were in need of ICU admission ranged from 0.80 to 0.86 in adults and 0.66 to 0.91 in children. Specificity ranged from 0.84 to 0.91 in adults and 0.82 to 0.87 in children (Table 3). This performance varied considerably between the different settings. Overall performance as indicated by the diagnostic odds ratio was lower in children than in adults except in the Maasstad hospital. However, the absolute number of children admitted to ICU in this hospital was very small.

**Table 3:** Diagnostic performance of the MTS for the identification of patients who died at the emergency department or required ICU admission.

	Erasmus MC		Maasstad		Fernando Fonseca	
	<16 years n = 6185	≥16 years n = 19,398	<16 years n = 7032	≥16 years n = 25,500	<16 years n = 52,843	≥16 years n = 177,705
Total ICU admissions, n (%)	148 (2.4)	333 (1.7%)	11 (0.2%)	266 (1.0%)	132 (0.2%)	403 (0.2%)
*Diagnostic accuracy (95% confidence interval)*						
Sensitivity	0.66 (0.58 to 0.73)	0.80 (0.76 to 0.84)	0.91 (0.62 to 0.98)	0.86 (0.81 to 0.90)	0.77 (0.69 to 0.83)	0.84 (0.80 to 0.87)
Specificity	0.87 (0.86 to 0.88)	0.91 (0.91 to 0.92)	0.83 (0.82 to 0.84)	0.85 (0.84 to 0.85)	0.82 (0.82 to 0.83)	0.84 (0.83 to 0.84)
Positive Likelihood Ratio	4.92 (4.30 to 5.62)	9.10 (8.48 to 9.75)	5.26 (4.34 to 6.39)	5.67 (5.36 to 6.00)	4.33 (3.94 to 4.77)	5.12 (4.90 to 5.35)
Negative Likelihood Ratio	0.40 (0.32 to 0.50)	0.21 (0.17 to 0.27)	0.11 (0.02 to 0.71)	0.16 (0.12 to 0.22)	0.29 (0.21 to 0.39)	0.19 (0.15 to 0.24)
Diagnostic Odds Ratio	12.4 (8.7 to 17.5)	42.5 (32.2 to 55.9)	47.9 (6.1 to 374.4)	34.6 (24.4 to 49.0)	15.2 (10.2 to 22.7)	27.0 (20.6 to 35.2)

When using the predefined 3-category reference classification, the MTS agreed with the reference standard in 61.6% of adult patients in the Erasmus MC, 49.7% in Maasstad Hospital and 51.7% in the Fernando Fonseca Hospital. In children, these percentages were 50.2%, 46.0% and 59.6% respectively. Overtriage was much more common than undertriage with percentages ranging from 26.9% to 44.0% in adults and 36.9% to 50.3% in children. Undertriage was present in 6.2% to 14.1% of adults and 3.5% to 5.8% of children.

Sensitivity to detect high urgent patients was moderate in the two Dutch hospitals and good in the Fernando Fonseca while specificity was good in all three hospitals. A summary of all diagnostic performance measures are presented in Table 4. The numbers of correct, over- and undertriage per MTS category are presented in the Supporting information (Tables A-F in S1 File)

**Table 4:** Diagnostic performance of the MTS, as determined by the 3-category reference standard.

	Erasmus MC		Maasstad		Fernando Fonseca	
	<16 years n = 6185	≥16 years n = 19,398	<16 years n = 7032	≥16 years n = 25,500	<16 years n = 52,843	≥16 years n = 177,705
						
*Absolute classification (%)*						
Correct triage	3104 (50.2)	11,940 (61.6)	3232 (46.0)	12,685 (49.7)	31,506 (59.6)	91,796 (51.7)
Overtriage	2722 (44.0)	5221 (26.9)	3534 (50.3)	11,228 (44.0)	19,487 (36.9)	60,928 (34.3)
Undertriage	359 (5.8)	2237 (11.5)	266 (3.8)	1587 (6.2)	1850 (3.5)	24,981 (14.1)
						
*Diagnostic accuracy (95% confidence interval)*						
Sensitivity	0.65 (0.61 to 0.70)	0.47 (0.44 to 0.49)	0.66 (0.57 to 0.74)	0.72(0.70 to 0.75)	0.83 (0.79 to 0.87)	0.87(0.85 to 0.90)
Specificity	0.89 (0.88 to 0.90)	0.94 (0.94 to 0.94)	0.83(0.83 to 0.84)	0.87 (0.87 to 0.87)	0.83 (0.82 to 0.83)	0.84 (0.84 to 0.84)
Positive likelihood ratio	6.12 (5.54 to 6.78)	7.66 (7.11 to 8.26)	3.99 (3.47 to 4.59)	5.59 (5.33 to 5.86)	4.79 (4.55 to 5.05)	5.36 (5.20 to 5.52)
Negative likelihood ratio	0.39 (0.34 to 0.44)	0.57 (0.55 to 0.59)	0.41 (0.32 to 0.52)	0.32 (0.29 to 0.35)	0.20 (0.16 to 0.26)	0.15(0.13 to 0.18)
Diagnostic Odds Ratio	15.8(12.8 to 19.6)	13.5(12.1 to 15.0)	9.8(6.7 to 14.5)	17.7(15.5 to 20.1)	23.8(17.7 to 32.0)	35.3 (28.4 to 43.9)

Sensitivity analyses showed that the modifications for children with fever improved performance in both settings. Without the modifications, the MTS would have had a slightly higher sensitivity at the cost of a lower specificity in the Erasmus MC, while in the hospital Fernando Fonseca sensitivity would be similar with a lower specificity (Tables A and B in S2 File).

### Performance in different age groups

**Fig 1:**
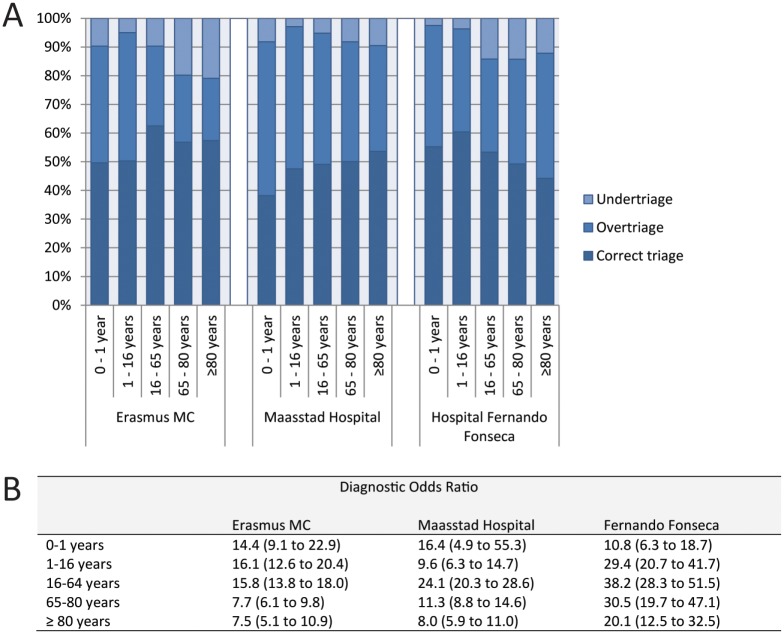
Performance of the MTS in different age groups. A) percentages under-, over-, and correct triage; B) diagnostic odds ratio’s.

Performance of the MTS in different age groups showed a large variation between settings (Fig 1; Tables A-C in S3 File). Overall, the diagnostic odds ratio was lower in elderly patients, aged 65 or older, when compared to the group of adults aged 16 to 65 and this was more prominent in the patients above the age of 80. While in all three hospitals specificity was lower in the older age groups, sensitivities varied when compared to adult patients.

Children had lower diagnostic odds ratios than the adult groups, except in the Erasmus MC. More specifically, specificity was lower in children compared to adults, but sensitivities varied compared to the adult reference group. There was no clear trend towards a decreased performance of the MTS in the youngest children.

### Performance of different flowcharts and discriminators

In adults, the most commonly used flowcharts in the three hospitals were “Limb problems”, “Unwell adult”, “Abdominal pain in adults”, “Chest pain”, “Shortness of breath in adults” and “Headache”, together accounting for 39% of adult patients. The general discriminators most often used were the consciousness and temperature discriminators, together accounting for 3.2% of adult patients. In hospital Fernando Fonseca, relatively few patients were triaged as high urgent and therefore performance could not be assessed for all flowcharts and discriminators.

**Fig 2:**
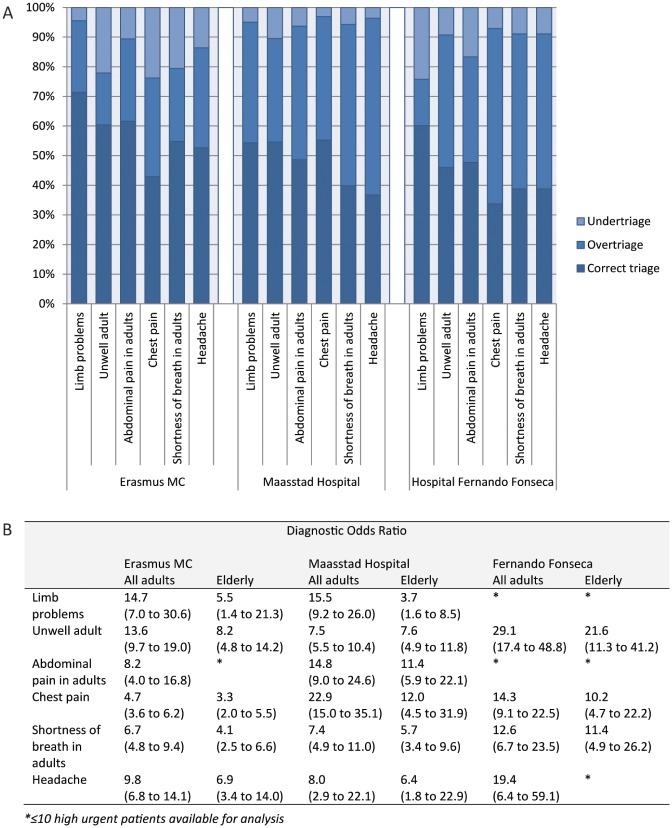
Performance of most commonly used MTS flowcharts. A) Percentages under-, over-, and correct triage; B) Diagnostic odds ratio’s.

**Fig 3:**
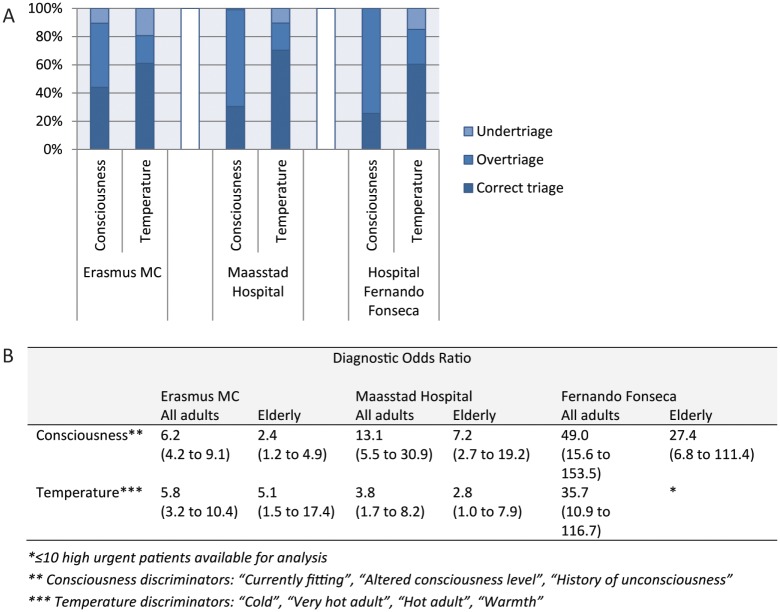
Performance of most commonly used MTS discriminators. A) Percentages under-, over-, and correct triage; B) Diagnostic odds ratio’s.

Overall, there was a large variation between settings in performance of the flowcharts and discriminators (Figs 2 and 3; Tables A-F in S4 File, and Tables A and B in S5 File) although performance in general was best in the hospital Fernando Fonseca and poorest in the Erasmus MC. In particular, sensitivities of the flowcharts and discriminators were very low. The flowcharts “Limb problems”, “Unwell adult”, “Abdominal pain in adults” and “Chest pain” even had sensitivities below the value of 0.5. The temperature discriminators had in all settings low sensitivities with high specificities, while consciousness discriminators had better sensitivities with moderate specificities.

Overall, there was a lower performance of the most commonly used flowcharts and discriminators in the elderly patients.

## Discussion

This multicenter observational study demonstrates that validity of the MTS for emergency department triage is moderate to good. When compared to a predefined, 3-category reference standard, sensitivity was 0.47 to 0.87 and specificity 0.84 to 0.94 for the triage of adult patients while sensitivity was 0.65 to 0.83 and specificity 0.83 to 0.89 for the triage of children. In all three hospitals, overall validity as determined by the diagnostic odds ratio was lowest in the youngest and oldest patients. One of the most remarkable findings was the high variability in performance of the MTS between the different emergency departments.

Previous studies have assessed performance of the MTS, the majority by evaluating associations between MTS triage category and hospitalization or resource use.[7, 8, 10, 16] Our study shows that specificity of the MTS when compared to a 3-category reference standard was very good, but sensitivity was moderate in two of the three hospitals. A low sensitivity indicates that high urgent patients are being “missed” by the triage system, which leads to longer waiting times for these patients and poses them at risk for adverse outcomes due to harm by delay in treatment. In our study, validity of the MTS for the most urgent patients, i.e. those requiring ICU admission, was better, but in absolute numbers the MTS still classifies 14 to 20% of adults and 9 to 34% of children in need of ICU admission as low urgent. These results indicate that improvement of the MTS is still needed.

Importantly, we found that performance of the MTS was lowest in the young and elderly patients. To our knowledge, this is the first study that assesses performance of the MTS for specific age groups. Only one study on the triage of patients with acute myocardial infarction specifically looked at age and found that patients above the age of 70 were less often correctly triaged as high urgent by the MTS.[13] There is also some evidence from the Emergency Severity Index and several trauma triage systems that elderly patients are at risk of undertriage.[25–28] Previous modifications targeted at children have been shown to improve validity of the MTS and were consequently partially implemented in the most recent MTS edition.[6, 19] Likewise, modifications aimed at elderly might be a promising way to improve triage for this patient group.

Our results show a remarkable variation between the three hospitals and we believe this can be explained by several factors. First, the differences in patient population attending the different emergency departments is likely to influence the validity of triage systems. It is well known that population characteristics, including demographic features, disease severity and disease prevalence influence the performance of diagnostic tests.[29] In the case of triage, it can be expected that increased patient complexity contributes to lower performance of a triage system because patients with rare disorders or multiple comorbid conditions may be more difficult to triage.[14] This could explain the lower performance of the MTS in the Erasmus MC, which is a tertiary hospital receiving relatively large numbers of complex patients. It is also possible, that disease prevalence plays a role and nurses apply triage criteria more strictly in settings with a lower prevalence of urgent patients, compared to settings with a higher prevalence. Secondly, some of the differences in performance of the MTS can be explained by the differences in availability of the reference standard items. The hospital Fernando Fonseca did not record information on high care admission, and emergency surgery, so misclassification of the outcome in some of the high urgent patients might have led to an overestimation of the validity of the MTS in this hospital. Moreover, it is possible that potential differences in clinical practice and different indications for reference standard items such as hospitalization and intravenous medication can explain some of the variability in the results. Nevertheless, this is probably not the entire explanation, as we also observed differences when using ICU admission as the reference standard, while we consider indications for ICU admission approximately similar in the three settings. Modifications of the MTS in the different hospitals may only have influenced the results marginally. Differences in MTS version between the hospitals were minor in adults, and in children only had a moderate impact on sensitivity and specificity in the Erasmus MC.

Moreover, we do not believe that differences in application of the MTS by the triage nurses have caused this large variation in results. Nurses in all three hospitals receive formal training in the MTS before they are allowed to triage patients, and previous studies, performed in different settings, showed that interrater reliability of the MTS was moderate to good.[10, 30–32] Even though a combination of patient and hospital related factors might contribute to the variability in performance of the MTS, this is simply a reflection of clinical practice. Variability in emergency department size, population and practices throughout the world simply exist, and triage needs to be conducted in any of these circumstances. Future multicenter studies should therefore focus on unravelling the factors that explain variability in triage performance between clinical settings. Consequently, it would be important to determine whether and how triage systems can be adapted to the local circumstances. Until then, our study indicates that the results of single-center studies evaluating a triage system should be interpreted with caution.

Our study has several strengths: it is based on a large cohort of almost 300,000 emergency department visits and it includes data from three different clinical settings, which increases generalizability. Moreover, we had less than 6% missing data on triage and reference standard items and we therefore believe risk of selection bias is low.

We assessed validity of the MTS by a 3-category reference standard as a proxy for true patient urgency, developed by a panel with expertise in the field of emergency care, and consisting of items undoubtedly related to patient urgency. In the absence of a golden standard for the evaluation of triage systems, the combination of multiple items to construct a reference standard is a valid approach.[33] Previous studies have evaluate triage systems with several single outcome measures such as hospitalization or resource use, which can merely be used to display trends in a certain outcome over different urgency categories. None of these individual items is able to perfectly distinguish the high from the low urgent patients. The combination of different items is a more precise way to describe true patient urgency and enables the evaluation of modifications to different triage categories. Still, our reference standard is a proxy of true patient urgency and therefore our results represent an estimation of the validity of the MTS.

A limitation of our study is that one of the hospitals did not have information on high care admission or emergency surgery, two items in the reference standard. This difference makes the interpretation of the results more difficult. Therefore, we also assessed ICU admission as another reference standard, which was collected in a similar way in all three settings. Moreover, due to the observational design of the study, based on routine data, we have to accept the occurrence of missing data, particularly in the documentation of vital signs. We assumed vital signs that were not measured to be normal, and although this is in line with clinical experience, and may be true for the majority of patients, we cannot exclude that we misclassified a small proportion of patients with abnormal vital signs that were not recorded.

Although our study has a large sample size, we still had insufficient numbers of high urgent patients available to derive and validate modifications for specific patient subgroups. This was also the case for the assessment of validity for specific flowcharts and discriminators, indicating that future studies should include at least a substantial number of high urgent patients per subgroup, specific flowchart or discriminator.

## Conclusion

This study shows that validity of the MTS is moderate to good, with poorer performance in the most vulnerable patient populations: the young and elderly. Moreover, the study reveals that it matters where you validate a triage system, since results are highly variable between the different clinical settings. Due to the large variability between the emergency departments, we could not propose modifications to improve the MTS. Future research should therefore be restricted to large multicenter studies, and conducted in diverse hospitals. This way, potential modifications to improve the MTS can be defined, and results can be generalized or adapted to different clinical settings.

## Supporting Information

S1 Table Differences between the MTS versions used in the three settings. (DOCX)

S2 Table Raw data. (SAV)

S1 Fig Flow diagram of the study population. (DOCX)

S1 File Numbers of correct, over- and undertriage per MTS category. (DOCX)

S2 File Sensitivity analysis of MTS performance, comparing validity of the MTS with and without modifications for children with fever. (DOCX)

S3 File Diagnostic performance of the MTS for different age groups, as determined by the 3-category reference standard. (DOCX)

S4 File Diagnostic performance of the MTS for the most commonly used MTS flowchart, as determined by the 3-category reference standard. (DOCX)

S5 File Diagnostic performance of the MTS for the most commonly used MTS discriminators, as determined by the 3-category reference standard. (DOCX)
