# Supplementary material for: Validity of the Manchester Triage System in emergency care: A prospective observational study
Source: PLoS One. 2017 Feb 2;12(2):e0170811. doi: 10.1371/journal.pone.0170811 (PMC5289484; doi:10.1371/journal.pone.0170811)
Supplement: S1 Table — (DOCX) [file pone.0170811.s001.docx]

**S1 Table. Differences between the MTS versions used in the three settings**

| Erasmus MC (based on the official Dutch translation of MTS version 2) [1] | | |
| --- | --- | --- |
| *Modification type* | *Original* | *New* |
| Altered urgency for subgroup  Altered urgency for subgroup   Altered urgency for subgroup  Altered urgency for subgroup  Altered urgency   Altered urgency | “Hot child” (*very urgent)*  “Persistent vomiting” (*urgent*)  “Not feeding” (*urgent*)  “Prolonged or interrupted crying” (*urgent*)  “Recent problem” (*standard*)  “Wheeze” (*standard*) | - “Hot child <3 months” (*very urgent*) - “Hot child >3 months” (*urgent*)  - “Febrile child” (*very urgent*), only in the neurologic flowcharts  - “Persistent vomiting < 3 months” (*urgent*)  - “Persistent vomiting >3 months” (*standard*)  - “Not feeding <1 year” (*urgent*)  - “Not feeding >1 year” (*standard*)  - “Prolonged or interrupted crying <1 year” (*urgent*)  - “Prolonged or interrupted crying >1 year” (*standard*)  - “Recent problem”, *standard* or *non-urgent* depending on the flowchart  “Wheeze” (*urgent*) |
| Maasstad Hospital (based on the official Dutch translation of MTS version 2) [1] | | |
| *No differences compared to original MTS* | | |
| Hospital Fernando Fonseca (based on the official Portuguese translation of MTS version 2) [2] | | |
| *Modification* | *Original* | *New* |
| Altered urgency for subgroup    New discriminator   New discriminator  New discriminator  New discriminator  New discriminator  New discriminator   New discriminator  New discriminator  New discriminator | “Hot child” (*very urgent*)  -  -  -  -    -  -    -    -    - | - Criança muito quente (*very urgent)*, if temperature ≥39.9°C  - Criança quente (*urgent*), if temperature 38.5-39.8°C  “Epigastric pain” (*very urgent*)  added to flowcharts Abdominal pain in adults and Shortness of breath in adults  “Bleeding disorder” (*urgent*)  added to several flowcharts  “History of head injury” (*urgent*) added to flowcharts Collapsed adult, Major trauma and Headache  “Lethargic” (*very urgent*) added to flowchart Diarrhoea and vomiting  “History of fitting” (*urgent*) added to flowchart Fits  “Hemoptise”(*very urgent*) added to flowchart Shortness of breath in adults, Shortness of breath in children and Unwell adult  “History of hemoptise” (*urgent*) added to flowcharts Shortness of breath in adults, Shortness of breath in children and Unwell adult  “Abnormal pulse” (*very urgent*) added to flowchart Unwell adult  “Recent trauma” (*urgent*) added to flowchart Wounds |

*1. Manchester Triage Groep. Triage voor de spoedeisende hulp. 2 ed: Elsevier gezondheidszorg; 2007.*

*2. Grupo Português de Triagem. Triagem no serviço de urgência. Amadora: Blackwell Publishing Ltd; 2009.*
