# Supplementary material for: Validity of the Manchester Triage System in emergency care: A prospective observational study
Source: PLoS One. 2017 Feb 2;12(2):e0170811. doi: 10.1371/journal.pone.0170811 (PMC5289484; doi:10.1371/journal.pone.0170811)
Supplement: S1 Fig — (DOCX) [file pone.0170811.s003.docx]

**S2 Fig. Flow diagram of the study population**

**ERASMUS
Medical Center**

**MAASSTAD
hospital**

**FERNANDO FONSECA hospital**

ED visits during study period

n=29,347

Included patients
n=25,583

ED visits during study period

n=33,604

ED visits during study period

n=243,139

Included patients
n=32,532

Included patients
n=230,548

Excluded (5.2%)
- Missing age and gender (n=5)

- Missing MTS urgency (n=1512)

- Missing disposition (n=11,074)

Excluded (3.2%)

- Missing age and gender (n=0)

- Missing MTS urgency (n=798)

- Missing disposition (n-274)

Excluded (12.8%)

- Missing age and gender (n=0)

- Missing MTS urgency (n=3462)
- Missing disposition (n=302)
