# Supplementary material for: Validity of the Manchester Triage System in emergency care: A prospective observational study
Source: PLoS One. 2017 Feb 2;12(2):e0170811. doi: 10.1371/journal.pone.0170811 (PMC5289484; doi:10.1371/journal.pone.0170811)
Supplement: S1 File — (DOCX) [file pone.0170811.s004.docx]

**S1 File. Numbers of correct, over- and undertriage per MTS category**

*Table A. Erasmus MC, <16 years*

|  | **Reference category 1** | **Reference category 2** | **Reference category 3** | **Total** |
| --- | --- | --- | --- | --- |
| Immediate | 99 | 16 | 11 | 126 |
| Very urgent | 189 | 268 | 319 | 776 |
| Urgent | 113 | 599 | 2108 | 2820 |
| Standard | 37 | 193 | 2061 | 2291 |
| Non-urgent | 2 | 14 | 156 | 172 |
| Total | 440 | 1090 | 4655 | 6185 |

☐ correct triage

☐ overtriage

☐ undertriage

*Table B. Maasstad, <16 years*

|  | **Reference category 1** | **Reference category 2** | **Reference category 3** | **Total** |
| --- | --- | --- | --- | --- |
| Immediate | 4 | 9 | 2 | 15 |
| Very urgent | 74 | 530 | 604 | 1208 |
| Urgent | 30 | 661 | 2389 | 3080 |
| Standard | 10 | 224 | 2475 | 2709 |
| Non-urgent | 0 | 2 | 18 | 20 |
| Total | 118 | 1426 | 5488 | 7032 |

☐ correct triage

☐ overtriage

☐ undertriage

*Table C. Fernando Fonseca, <16 years*

|  | **Reference category 1** | **Reference category 2** | **Reference category 3** | **Total** |
| --- | --- | --- | --- | --- |
| Immediate | 21 | 135 | 80 | 236 |
| Very urgent | 245 | 1488 | 7437 | 9170 |
| Urgent | 28 | 1516 | 10347 | 11891 |
| Standard | 24 | 1778 | 29072 | 30874 |
| Non-urgent | 1 | 19 | 652 | 672 |
| Total | 319 | 4936 | 47588 | 52843 |

☐ correct triage

☐ overtriage

☐ undertriage

*Table D. Erasmus MC, ≥16 years*

|  | **Reference category 1** | **Reference category 2** | **Reference category 3** | **Total** |
| --- | --- | --- | --- | --- |
| Immediate | 256 | 43 | 7 | 306 |
| Very urgent | 635 | 779 | 235 | 1649 |
| Urgent | 890 | 3649 | 4157 | 8696 |
| Standard | 130 | 1212 | 7383 | 8725 |
| Non-urgent | 0 | 5 | 17 | 22 |
| Total | 1911 | 5688 | 11799 | 19398 |

☐ correct triage

☐ overtriage

☐ undertriage

*Table E. Maasstad, ≥16 years*

|  | **Reference category 1** | **Reference category 2** | **Reference category 3** | **Total** |
| --- | --- | --- | --- | --- |
| Immediate | 143 | 40 | 10 | 193 |
| Very urgent | 773 | 2020 | 1074 | 3867 |
| Urgent | 313 | 5334 | 8084 | 13731 |
| Standard | 35 | 1227 | 6361 | 7623 |
| Non-urgent | 0 | 12 | 74 | 86 |
| Total | 1264 | 8633 | 15603 | 25500 |

☐ correct triage

☐ overtriage

☐ undertriage

*Table F. Fernando Fonseca, ≥16 years*

|  | **Reference category 1** | **Reference category 2** | **Reference category 3** | **Total** |
| --- | --- | --- | --- | --- |
| Immediate | 94 | 939 | 96 | 1129 |
| Very urgent | 538 | 19654 | 8140 | 28332 |
| Urgent | 76 | 32711 | 32099 | 64886 |
| Standard | 15 | 24105 | 54962 | 79082 |
| Non-urgent | 1 | 784 | 3491 | 4276 |
| Total | 724 | 78193 | 98788 | 177705 |

☐ correct triage

☐ overtriage

☐ undertriage
