# Supplementary material for: Validity of the Manchester Triage System in emergency care: A prospective observational study
Source: PLoS One. 2017 Feb 2;12(2):e0170811. doi: 10.1371/journal.pone.0170811 (PMC5289484; doi:10.1371/journal.pone.0170811)
Supplement: S2 File — (DOCX) [file pone.0170811.s005.docx]

**S2 File. Sensitivity analysis of MTS performance, comparing validity of the MTS with and without modifications for children with fever**

*Table A. Reference standard: mortality or ICU admission*

|  | Erasmus MC (n=6185) | | Fernando Fonseca (n=52,843) | |
| --- | --- | --- | --- | --- |
|  | MTS including modifications | MTS simulated without modifications | MTS including modifications | MTS simulated without modifications |
|  | | | | |
| *Diagnostic accuracy (95% confidence interval)* | | | | |
| Sensitivity | 0.66  (0.58 to 0.73) | 0.68  (0.60 to 0.75) | 0.77  (0.69-0.83) | 0.77  (0.69-0.83) |
| Specificity | 0.87  (0.86 to 0.88) | 0.77  (0.76 to 0.78) | 0.82  (0.82-0.83) | 0.80  (0.80-0.81) |
| Positive Likelihood Ratio | 4.92  (4.30 to 5.62) | 3.02  (2.68 to 3.41) | 4.33  (3.94-4.77) | 3.87  (3.52-4.26) |
| Negative Likelihood Ratio | 0.40  (0.32 to 0.50) | 0.41  (0.32 to 0.52) | 0.29  (0.21-0.39) | 0.29  (0.22-0.40) |
| Diagnostic Odds Ratio | 12.4  (8.7 to 17.5) | 7.4  (5.2 to 10.5) | 15.2  (10.2-22.7) | 13.2  (8.9-19.8) |

*Table B. Reference standard: the 3-category reference classification*

|  | Erasmus MC (n=6185) | | Fernando Fonseca (n=52,843) | |
| --- | --- | --- | --- | --- |
|  | MTS including modifications | MTS simulated without modifications | MTS including modifications | MTS simulated without modifications |
|  | | | | |
| *Absolute classification (%)* | | | | |
| Correct triage | 3104 (50.2) | 3004 (48.6) | 31,506 (59.6) | 31,395 (59.4) |
| Overtriage | 2722 (44.0) | 2853 (46.1) | 19,487 (36.9) | 19,599 (37.1) |
| Undertriage | 359 (5.8) | 328 (5.3) | 1850 (3.5) | 1849 (3.5) |
|  | | | | |
| *Diagnostic accuracy (95% confidence interval)* | | | | |
| Sensitivity | 0.65  (0.61 to 0.70) | 0.72  (0.68 to 0.76) | 0.83  (0.79 to 0.87) | 0.84  (0.79 to 0.87) |
| Specificity | 0.89  (0.88 to 0.90) | 0.80  (0.79 to 0.81) | 0.83  (0.82 to 0.83) | 0.80  (0.80 to 0.81) |
| Positive Likelihood Ratio | 6.12  (5.54 to 6.78) | 3.61  (3.34 to 3.90) | 4.79  (4.55 to 5.05) | 4.29  (4.08 to 4.52) |
| Negative Likelihood Ratio | 0.39  (0.34 to 0.44) | 0.35  (0.30 to 0.41) | 0.20  (0.16 to 0.26) | 0.20  (0.16 to 0.26) |
| Diagnostic Odds Ratio | 15.8  (12.8 to 19.6) | 10.3  (8.3 to 12.9) | 23.8  (17.7 to 32.0) | 21.2  (15.7 to 28.5) |
