# Supplementary material for: Validity of the Manchester Triage System in emergency care: A prospective observational study
Source: PLoS One. 2017 Feb 2;12(2):e0170811. doi: 10.1371/journal.pone.0170811 (PMC5289484; doi:10.1371/journal.pone.0170811)
Supplement: S3 File — (DOCX) [file pone.0170811.s006.docx]

**S3 File. Diagnostic performance of the MTS for different age groups, as determined by the 3-category reference standard**

*Table A. Erasmus MC*

| **Erasmus MC** | | | | | |
| --- | --- | --- | --- | --- | --- |
|  | 0-1 years  n=1107 | 1-16 years n=5078 | 16-65 years  n=15,980 | 65-80 years  n=2598 | ≥ 80 years n=820 |
|  |  |  |  |  |  |
| *Absolute classification (%)* | | | | | |
| Correct triage | 549 (49.6) | 2555 (50.3) | 9994 (62.5) | 1475 (56.8) | 471 (57.4) |
| Overtriage | 451 (40.7) | 2271 (44.7) | 4435 (27.8) | 608 (23.4) | 178 (21.7) |
| Undertriage | 107 (9.7) | 252 (5.0) | 1551 (9.7) | 515 (19.8) | 171 (20.9) |
|  |  |  |  |  |  |
| *Diagnostic accuracy (95% confidence interval)* | | | | | |
| Sensitivity | 0.69  (0.60 to 0.77) | 0.64  (0.59 to 0.69) | 0.48  (0.45 to 0.50) | 0.43  (0.39 to 0.48) | 0.49  (0.42 to 0.56) |
| Specificity | 0.86  (0.84 to 0.88) | 0.90  (0.89 to 0.91) | 0.95  (0.94 to 0.95) | 0.91  (0.90 to 0.92) | 0.89  (0.86 to 0.91) |
| Positive Likelihood Ratio | 5.13  (4.18 to 6.28) | 6.38  (5.68 to 7.16) | 8.75  (8.01 to 9.57) | 4.82  (4.07 to 5.71) | 4.30  (3.30 to 5.59) |
| Negative Likelihood Ratio | 0.35  (0.26 to 0.48) | 0.40  (0.34 to 0.46) | 0.55  (0.53 to 0.58) | 0.62  (0.58 to 0.68) | 0.58  (0.50 to 0.67) |
| Diagnostic Odds Ratio | 14.4  (9.1 to 22.9) | 16.1  (12.6 to 20.4) | 15.8  (13.8 to 18.0) | 7.7  (6.1 to 9.8) | 7.5  (5.1 to 10.9) |

*Table B. Maasstad*

| **Maasstad** | | | | | |
| --- | --- | --- | --- | --- | --- |
|  | 0-1 years  n=1212 | 1-16 years n=5820 | 16-65 years  n=18,226 | 65 - 80 years  n=4494 | ≥ 80 years n=2780 |
|  |  |  |  |  |  |
| *Absolute classification (%)* | | | | | |
| Correct triage | 463 (38.2) | 2769 (47.6) | 8945 (49.1) | 2249 (50.0) | 1491 (53.6) |
| Overtriage | 650 (53.6) | 2884 (49.6) | 8327 (45.7) | 1875(41.7) | 1026 (36.9) |
| Undertriage | 99 (8.2) | 167 (2.9) | 954 (5.2) | 370 (8.2) | 263 (9.5) |
|  |  |  |  |  |  |
| *Diagnostic accuracy (95% confidence interval)* | | | | | |
| Sensitivity | 0.88  (0.69 to 0.96) | 0.61  (0.51 to 0.70) | 0.73  (0.70 to 0.77) | 0.74  (0.69 to 0.78) | 0.67  (0.60 to 0.73) |
| Specificity | 0.70  (0.67 to 0.73) | 0.86  (0.85 to 0.87) | 0.90  (0.89 to 0.90) | 0.80  (0.79 to 0.81) | 0.80  (0.78 to 0.81) |
| Positive Likelihood Ratio | 2.92  (2.45 to 3.48) | 4.40  (3.69 to 5.24) | 7.13  (6.70 to 7.58) | 3.73  (3.41 to 4.08) | 3.32  (2.93 to 3.76) |
| Negative Likelihood Ratio | 0.18  (0.06 to 0.52) | 0.46  (0.36 to 0.59) | 0.30  (0.26 to 0.33) | 0.33  (0.28 to 0.39) | 0.41  (0.34 to 0.51) |
| Diagnostic Odds Ratio | 16.4  (4.8 to 55.2) | 9.6  (6.3 to 14.7) | 24.1  (20.3 to 28.6) | 11.3  (8.8 to 14.6) | 8.0  (5.9 to 11.0) |

*Table C. Fernando Fonseca*

| **Fernando Fonseca** | | | | | |
| --- | --- | --- | --- | --- | --- |
|  | 0-1 years  n=8185 | 1-16 years n=44,658 | 16-65 years  n=127,562 | 65 - 80 years  n=32,689 | ≥ 80 years n=17,454 |
|  |  |  |  |  |  |
| *Absolute classification (%)* | | | | | |
| Correct triage | 4518 (55.2) | 26,988 (60.4) | 68,012 (53.3) | 16,071 (49.2) | 7713 (44.2) |
| Overtriage | 3465 (42.3) | 16,022 (35.9) | 41,376 (32.4) | 11,943 (36.5) | 7609 (43.6) |
| Undertriage | 202 (2.5) | 1648 (3.7) | 18,174 (14.2) | 4675 (14.3) | 2132 (12.2) |
|  |  |  |  |  |  |
| *Diagnostic accuracy (95% confidence interval)* | | | | | |
| Sensitivity | 0.81  (0.71 to 0.88) | 0.84  (0.79 to 0.88) | 0.86  (0.82 to 0.89) | 0.89  (0.84 to 0.92) | 0.89  (0.83 to 0.93) |
| Specificity | 0.72  (0.71 to 0.73) | 0.85  (0.84 to 0.85) | 0.86  (0.86 to 0.87) | 0.79  (0.79 to 0.80) | 0.72  (0.71 to 0.72) |
| Positive LR | 2.89  (2.59 to 3.23) | 5.45  (5.13 to 5.78) | 6.31  (6.04 to 6.60) | 4.32  (4.10 to 4.56) | 3.14  (2.96 to 3.33) |
| Negative LR | 0.27  (0.17 to 0.42) | 0.19  (0.14 to 0.25) | 0.17  (0.13 to 0.21) | 0.14  (0.10 to 0.21) | 0.16  (0.10 to 0.24) |
| DOR | 10.8  (6.3 to 18.7) | 29.4  (20.7 to 41.7) | 38.2  (28.3 to 51.5) | 30.5  (19.7 to 47.1) | 20.1  (12.5 to 32.5) |
