# Supplementary material for: Validity of the Manchester Triage System in emergency care: A prospective observational study
Source: PLoS One. 2017 Feb 2;12(2):e0170811. doi: 10.1371/journal.pone.0170811 (PMC5289484; doi:10.1371/journal.pone.0170811)
Supplement: S4 File — (DOCX) [file pone.0170811.s007.docx]

**S4 File. Diagnostic performance of the MTS for the most commonly used MTS flowchart, as determined by the 3-category reference standard**

*Table A. Flowchart Limb problems*

|  | | Erasmus MC | | | Maasstad | | | Fernando Fonseca | |
| --- | --- | --- | --- | --- | --- | --- | --- | --- | --- |
|  | All adults n=4021 | | Elderly n=415 | All adults n=6829 | | Elderly n=1431 | All adults n=24,723 | | Elderly n=6504 |
| High urgent patients, n (%) | 80 (2.0) | | 16 (3.9) | 144 (2.1) | | 63 (4.4) | 528 (2.1) | | 183 (2.8) |
| *Absolute classification (%)* | | | | | | | | | |
| Correct triage | 2870 (71.4) | | 253 (61.0) | 3705 (54.3) | | 722 (50.5) | 14,871 (60.2) | | 3551 (54.6) |
| Overtriage | 972 (24.2) | | 116 (28.0) | 2782 (40.7) | | 584 (40.8) | 3845 (15.6) | | 1061 (16.3) |
| Undertriage | 179 (4.5) | | 46 (11.1) | 342 (5.0) | | 125 (8.7) | 6007 (24.3) | | 1892 (29.1) |
|  |  | |  |  | |  |  | |  |
| *Diagnostic accuracy (95% confidence interval)* | | | | | | | | | |
| Sensitivity | 0.21  (0.12 to 0.34) | | 0.16 (0.06 to 0.38) | 0.22 (0.15 to 0.32) | | 0.13  (0.07 to 0.25) | * | | * |
| Specificity | 0.98  (0.98 to 0.99) | | 0.97 (0.94 to 0.98) | 0.98 (0.98 to 0.98) | | 0.96 (0.95 to 0.97) |  | |  |
| Positive LR | 11.82  (6.50 to 21.51) | | 4.81  (1.50 to 15.47) | 12.23 (8.07 to 18.54) | | 3.31 (1.59 to 6.92) |  | |  |
| Negative LR | 0.81  (0.70 to 0.93) | | 0.87  (0.72 to 1.06) | 0.79 (0.71 to 0.88) | | 0.90 (0.81 to 1.01) |  | |  |
| DOR | 14.7 (7.0 to 30.6) | | 5.5 (1.4 to 21.3) | 15.5 (9.2 to 25.9) | | 3.7 (1.6 to 8.5) |  | |  |

**≤10 high urgent patients available for analysis*

*Table B. Flowchart Unwell adult*

|  | | Erasmus MC | | | Maasstad | | | Fernando Fonseca | |
| --- | --- | --- | --- | --- | --- | --- | --- | --- | --- |
|  | All adults n=2429 | | Elderly n=672 | All adults n=2541 | | Elderly n=1254 | All adults n=13,272 | | Elderly n=6117 |
| High urgent patients, n (%) | 170 (7.0) | | 70 (10.4) | 463 (18.2) | | 263 (21.0) | 3507 (26.4) | | 2199 (35.9) |
| *Absolute classification (%)* | | | | | | | | | |
| Correct triage | 1467 (60.4) | | 388 (57.7) | 1387 (54.6) | | 742 (59.2) | 6101 (46.0) | | 2451 (40.1) |
| Overtriage | 426(17.5) | | 104 (15.5) | 887 (34.9) | | 357 (28.5) | 5934 (44.7) | | 3120 (51.0) |
| Undertriage | 536 (22.1) | | 180 (26.8) | 267 (10.5) | | 155 (12.4) | 1237 (9.3) | | 546 (8.9) |
|  |  | |  |  | |  |  | |  |
| *Diagnostic accuracy (95% confidence interval)* | | | | | | | | | |
| Sensitivity | 0.33  (0.28 to 0.39) | | 0.36  (0.27 to 0.47) | 0.58 (0.50 to 0.65) | | 0.62 (0.52 to 0.71) | 0.91  (0.86 to 0.94) | | 0.92  (0.86 to 0.96) |
| Specificity | 0.96  (0.96 to 0.97) | | 0.93  (0.91 to 0.95) | 0.85 (0.83 to 0.86) | | 0.82 (0.80 to 0.85) | 0.74  (0.74 to 0.75) | | 0.65  (0.64 to 0.66) |
| Positive LR | 9.42 (7.14 to 12.42) | | 5.59  (3.70 to 8.45) | 3.77 (3.22 to 4.42) | | 3.53 (2.89 to 4.31) | 3.56  (3.37 to 3.76) | | 2.65  (2.49 to 2.82) |
| Negative LR | 0.69  (0.64 to 0.75) | | 0.68  (0.58 to 0.80) | 0.50 (0.42 to 0.59) | | 0.46 (0.36 to 0.60) | 0.12  (0.08 to 0.20) | | 0.12  (0.07 to 0.22) |
| DOR | 13.6 (9.7 to 19.0) | | 8.2 (4.8 to 14.2) | 7.5 (5.5 to 10.4) | | 7.6 (4.9 to 11.8) | 29.1  (17.4 to 48.8) | | 21.6  (11.3 to 41.2) |

*Table C. Flowchart Abdominal pain in adults*

|  | | Erasmus MC | | | Maasstad | | | Fernando Fonseca | |
| --- | --- | --- | --- | --- | --- | --- | --- | --- | --- |
|  | All adults n=2027 | | Elderly n=328 | All adults n=3310 | | Elderly n=656 | All adults n=13,872 | | Elderly n=3107 |
| High urgent patients, n (%) | 91 (4.5) | | 21 (6.4%) | 327 (9.9) | | 106 (16.2) | 2302 (16.6) | | 507 (16.3) |
| *Absolute classification (%)* | | | | | | | | | |
| Correct triage | 1248 (61.6) | | 231 (70.4) | 1610 (48.6) | | 387 (59.0) | 6622(47.7) | | 1593 (51.3) |
| Overtriage | 564 (27.8) | | 57 (17.4) | 1491 (45.0) | | 207 (31.6) | 4938 (35.6) | | 861 (27.7) |
| Undertriage | 215 (10.6) | | 40 (12.2) | 209 (6.3) | | 62 (9.5) | 2312 (16.7) | | 653 (21.0) |
|  |  | |  |  | |  |  | |  |
| *Diagnostic accuracy (95% confidence interval)* | | | | | | | | | |
| Sensitivity | 0.26  (0.15 to 0.40) | | * | 0.59 (0.47 to 0.70) | | 0.63 (0.48 to 0.76) | * | | * |
| Specificity | 0.96  (0.95 to 0.97) | |  | 0.91 (0.90 to 0.92) | | 0.87 (0.84 to 0.90) |  | |  |
| Positive LR | 6.34  (3.65 to 11.03) | |  | 6.66 (5.29 to 8.37) | | 4.87 (3.58 to 6.63) |  | |  |
| Negative LR | 0.78  (0.65 to 0.92) | |  | 0.45 (0.34 to 0.60) | | 0.43 (0.29 to 0.63) |  | |  |
| DOR | 8.2 (4.0 to 16.8) | |  | 14.8 (8.9 to 24.6) | | 11.4 (5.9 to 22.1) |  | |  |

**≤10 high urgent patients available for analysis*

*Table D. Flowchart Chest pain*

|  | | Erasmus MC | | | Maasstad | | | Fernando Fonseca | |
| --- | --- | --- | --- | --- | --- | --- | --- | --- | --- |
|  | All adults n=1349 | | Elderly n=373 | All adults n=927 | | Elderly n=220 | All adults n=12,403 | | Elderly n=3968 |
| High urgent patients, n (%) | 285 (21.1) | | 95 (25.5) | 608 (65.6) | | 175 (79.5) | 4781 (38.5) | | 1982 (49.9) |
| *Absolute classification (%)* | | | | | | | | | |
| Correct triage | 582 (43.1) | | 159 (42.6) | 512 (55.2) | | 129 (58.6) | 4182 (33.7) | | 1122 (28.3) |
| Overtriage | 452 (33.5) | | 89 (23.9) | 386 (41.6) | | 86 (39.1) | 7343 (59.2) | | 2631 (66.3) |
| Undertriage | 315 (23.4) | | 125 (33.5) | 29 (3.1) | | 5 (2.3) | 878 (7.1) | | 215 (5.4) |
|  |  | |  |  | |  |  | |  |
| *Diagnostic accuracy (95% confidence interval)* | | | | | | | | | |
| Sensitivity | 0.39  (0.34 to 0.43) | | 0.37  (0.30 to 0.44) | 0.94 (0.91 to 0.96) | | 0.95 (0.90 to 0.98) | 0.90  (0.85 to 0.93) | | 0.91  (0.82 to 0.95) |
| Specificity | 0.88  (0.86 to 0.90) | | 0.85  (0.80 to 0.90) | 0.60 (0.56 to 0.65) | | 0.36 (0.28 to 0.45) | 0.62  (0.61 to 0.63) | | 0.51  (0.49 to 0.52) |
| Positive LR | 3.27  (2.64 to 4.05) | | 2.48  (1.68 to 3.68) | 2.37 (2.12 to 2.66) | | 1.49 (1.29 to 1.73) | 2.38  (2.26 to 2.51) | | 1.85  (1.71 to 2.00) |
| Negative LR | 0.69  (0.64 to 0.75) | | 0.74  (0.66 to 0.84) | 0.10 (0.07 to 0.15) | | 0.13 (0.05 to 0.31) | 0.17  (0.11 to 0.25) | | 0.18  (0.09 to 0.37) |
| DOR | 4.7 (3.6 to 6.2) | | 3.3 (2.0 to 5.5) | 22.9 (15.0 to 34.9) | | 11.7 (4.4 to 31.1) | 14.3  (9.1 to 22.5) | | 10.2  (4.7 to 22.2) |

*Table E. Flowchart Shortness of breath in adults*

|  | | Erasmus MC | | | Maasstad | | | Fernando Fonseca | |
| --- | --- | --- | --- | --- | --- | --- | --- | --- | --- |
|  | All adults n=1110 | | Elderly n=382 | All adults n=2312 | | Elderly n=1166 | All adults n=10,907 | | Elderly n=6408 |
| High urgent patients, n (%) | 237 (21.4) | | 101 (26.4) | 955 (41.3) | | 604 (51.8) | 4495 (41.2) | | 3252 (50.7) |
| *Absolute classification (%)* | | | | | | | | | |
| Correct triage | 608 (54.8) | | 198 (51.8) | 919 (39.7) | | 485 (41.6) | 4225 (38.7) | | 2118 (33.1) |
| Overtriage | 273 (24.6) | | 84 (22.0) | 1260 (54.5) | | 605 (51.9) | 5706 (52.3) | | 3796 (59.2) |
| Undertriage | 229 (20.6) | | 100 (26.2) | 133 (5.8) | | 76 (6.5) | 976 (8.9) | | 494 (7.7) |
|  |  | |  |  | |  |  | |  |
| *Diagnostic accuracy (95% confidence interval)* | | | | | | | | | |
| Sensitivity | 0.53  (0.46 to 0.60) | | 0.48 (0.39 to 0.58) | 0.82 (0.75 to 0.87) | | 0.84 (0.76 to 0.90) | 0.90  (0.82 to 0.94) | | 0.92  (0.84 to 0.96) |
| Specificity | 0.86  (0.83 to 0.88) | | 0.81  (0.76 to 0.86) | 0.62 (0.60 to 0.64) | | 0.52 (0.49 to 0.55) | 0.59  (0.58 to 0.60) | | 0.50  (0.49 to 0.51) |
| Positive LR | 3.68  (3.00 to 4.52) | | 2.59  (1.88 to 3.55) | 2.15 (1.97 to 2.35) | | 1.74 (1.58 to 1.93) | 2.20  (2.05 to 2.36) | | 1.83  (1.71 to 1.97) |
| Negative LR | 0.55  (0.47 to 0.64) | | 0.64  (0.53 to 0.78) | 0.29 (0.21 to 0.40) | | 0.31 (0.20 to 0.47) | 0.18  (0.10 to 0.31) | | 0.16  (0.08 to 0.35) |
| DOR | 6.7 (4.8 to 9.4) | | 4.1 (2.5 to 6.6) | 7.3 (4.9 to 10.9) | | 5.7 (3.4 to 9.6) | 12.6 (6.7 to 23.5) | | 11.4 (4.9 to 26.2) |

*Table F. Flowchart Headache*

| **Headache** | | Erasmus MC | | | Maasstad | | | Fernando Fonseca | |
| --- | --- | --- | --- | --- | --- | --- | --- | --- | --- |
|  | All adults n=889 | | Elderly n=163 | All adults n=730 | | Elderly n=273 | All adults n=7970 | | Elderly n=1874 |
| High urgent patients, n (%) | 194 (21.8) | | 62 (38.0) | 219 (30.0) | | 117 (42.9) | 1228 (15.4) | | 375 (20.0) |
| *Absolute classification (%)* | | | | | | | | | |
| Correct triage | 468 (52.6) | | 91 (55.8) | 268 (36.7) | | 116 (42.5) | 4017 (50.4) | | 859 (45.8) |
| Overtriage | 300 (33.7) | | 39 (23.9) | 436 (59.7) | | 145 (53.1) | 2856 (35.8) | | 797 (42.5) |
| Undertriage | 121 (13.6) | | 33 (20.2) | 26 (3.6) | | 12 (4.4) | 1097 (13.8) | | 218 (11.6) |
|  |  | |  |  | |  |  | |  |
| *Diagnostic accuracy (95% confidence interval)* | | | | | | | | | |
| Sensitivity | 0.56  (0.49 to 0.63) | | 0.62  (0.50 to 0.72) | 0.76 (0.55 to 0.89) | | 0.81 (0.57 to 0.93) | 0.78  (0.55 to 0.91) | | * |
| Specificity | 0.88  (0.86 to 0.91) | | 0.81  (0.72 to 0.88) | 0.71 (0.68 to 0.75) | | 0.60 (0.53 to 0.65) | 0.85  (0.84 to 0.86) | |  |
| Positive LR | 4.86  (3.82 to 6.18) | | 3.26  (2.05 to 5.19) | 2.66 (2.04 to 3.47) | | 2.01 (1.52 to 2.65) | 5.09  (3.96 to 6.56) | |  |
| Negative LR | 0.50  (0.42 to 0.58) | | 0.47  (0.35 to 0.64) | 0.33 (0.16 to 0.72) | | 0.31 (0.11 to 0.88) | 0.26  (0.11 to 0.62) | |  |
| DOR | 9.8 (6.8 to 14.1) | | 6.9 (3.4 to 14.0) | 8.0 (2.9 to 22.1) | | 6.4 (1.8 to 22.9) | 19.4  (6.4 to 59.1) | |  |

**≤10 high urgent patients available for analysis*
