# Supplementary material for: Validity of the Manchester Triage System in emergency care: A prospective observational study
Source: PLoS One. 2017 Feb 2;12(2):e0170811. doi: 10.1371/journal.pone.0170811 (PMC5289484; doi:10.1371/journal.pone.0170811)
Supplement: S5 File — (DOCX) [file pone.0170811.s008.docx]

**S5 File. Diagnostic performance of the MTS** **for the most commonly used MTS discriminators, as determined by the 3-category reference standard**

*Table A. Consciousness discriminators*

|  | | Erasmus MC | | | Maasstad | | | Fernando Fonseca | |
| --- | --- | --- | --- | --- | --- | --- | --- | --- | --- |
|  | All adults n=592 | | Elderly n=135 | All adults n=617 | | Elderly n=277 | All adults n=4451 | | Elderly n=2553 |
|  |  | |  |  | |  |  | |  |
| *Absolute classification (%)* | | | | | | | | | |
| Correct triage | 260 (43.9) | | 65 (48.1) | 188 (30.5) | | 104 (37.5) | 1133 (25.5) | | 530 (20.8) |
| Overtriage | 269 (45.4) | | 48 (35.6) | 423 (68.6) | | 168 (60.6) | 3315 (74.5) | | 2021 (79.2) |
| Undertriage | 63 (10.6) | | 22(16.3) | 6 (1.0) | | 5 (1.8) | 3 (0.1) | | 2 (0.1) |
|  |  | |  |  | |  |  | |  |
| *Diagnostic accuracy (95% confidence interval)* | | | | | | | | | |
| Sensitivity | 0.64  (0.57 to 0.71) | | 0.61  (0.48 to 0.73) | 0.90 (0.80 to 0.95) | | 0.86 (0.71 to 0.94) | 0.98  (0.96 to 0.99) | | 0.98  (0.94 to 1.00) |
| Specificity | 0.78  (0.73 to 0.81) | | 0.60  (0.49 to 0.70) | 0.59 (0.55 to 0.63) | | 0.55 (0.48 to 0.61) | 0.43  (0.42 to 0.45) | | 0.32  (0.31 to 0.34) |
| Positive LR | 2.87  (2.32 to 3.54) | | 1.54  (1.10 to 2.18) | 2.21 (1.94 to 2.52) | | 1.89 (1.55 to 2.29) | 1.73  (1.68 to 1.79) | | 1.46  (1.40 to 1.51) |
| Negative LR | 0.46  (0.38 to 0.57) | | 0.64  (0.44 to 0.93) | 0.17 (0.08 to 0.36) | | 0.26 (0.12 to 0.59) | 0.04  (0.01 to 0.11) | | 0.05  (0.01 to 0.21) |
| DOR | 6.2 (4.2 to 9.1) | | 2.4 (1.2 to 4.9) | 13.1 (5.5 to 30.9) | | 7.2 (2.7 to 19.2) | 49.0 (15.6 to 153.5) | | 27.4 (6.8 to 111.4) |

*Table B. Temperature discriminators*

|  | | Erasmus MC | | | Maasstad | | | Fernando Fonseca | |
| --- | --- | --- | --- | --- | --- | --- | --- | --- | --- |
|  | All adults n=788 | | Elderly n=217 | All adults n=441 | | Elderly n=195 | All adults n=2305 | | Elderly n=383 |
|  |  | |  |  | |  |  | |  |
| *Absolute classification (%)* | | | | | | | | | |
| Correct triage | 481 (61.0) | | 148 (68.2) | 310 (70.3) | | 147(75.4) | 1392 (60.4) | | 229 (59.8) |
| Overtriage | 155 (19.7) | | 30 (13.8) | 85 (19.3) | | 26 (13.3) | 570 (24.7) | | 102 (26.6) |
| Undertriage | 152 (19.3) | | 39 (18.0) | 46 (10.4) | | 22 (11.3) | 343 (14.9) | | 52 (13.6) |
|  |  | |  |  | |  |  | |  |
| *Diagnostic accuracy (95% confidence interval)* | | | | | | | | | |
| Sensitivity | 0.19  (0.13 to 0.27) | | 0.17  (0.07-0.34) | 0.19 (0.11 to 0.31) | | 0.21 (0.10 to 0.40) | 0.42  (0.19 to 0.68) | | * |
| Specificity | 0.96  (0.94 to 0.97) | | 0.96  (0.92 to 0.98) | 0.94 (0.91 to 0.96) | | 0.91 (0.86 to 0.94) | 0.98  (0.97 to 0.99) | |  |
| Positive LR | 4.87  (2.91 to 8.15) | | 4.45  (1.51 to 13.12) | 3.22 (1.66 to 6.25) | | 2.39 (1.01 to 5.63) | 21.23  (10.24 to 44.03) | |  |
| Negative LR | 0.84  (0.77 to 0.92) | | 0.87  (0.74 to 1.02) | 0.86 (0.75 to 0.98) | | 0.86 (0.71 to 1.05) | 0.60  (0.37 to 0.96) | |  |
| DOR | 5.8 (3.2 to 10.4) | | 5.1 (1.5 to 17.4) | 3.8 (1.7 to 8.2) | | 2.8 (1.0 to 7.9) | 35.7 (10.9 to 116.7) | |  |

**≤10 high urgent patients available for analysis*
